# The LncRNA CASC11 Promotes Colorectal Cancer Cell Proliferation and Migration by Adsorbing miR-646 and miR-381-3p to Upregulate Their Target RAB11FIP2

**DOI:** 10.3389/fonc.2021.657650

**Published:** 2021-04-15

**Authors:** Wei Zhang, Xiaomin Li, Wenjuan Zhang, Yanxia Lu, Weihao Lin, Lawei Yang, Zheying Zhang, Xuenong Li

**Affiliations:** ^1^Department of Pathology, School of Basic Medical Sciences, Southern Medical University, Guangzhou, China; ^2^Department of Pathology, The First Affiliated Hospital (Yijishan Hospital) of Wannan Medical College, Wuhu, China; ^3^Department of Pathology, Xinxiang Medical University, Xinxiang, China

**Keywords:** CASC11, RAB11FIP2, colorectal cancer, miR-381-3p, miR-646

## Abstract

**Background:**

We previously reported that the long non-coding RNA (lncRNA) CASC11 promotes colorectal cancer (CRC) progression as an oncogene by binding to HNRNPK. However, it remains unknown whether CASC11 can act as a competitive endogenous RNA (ceRNA) in CRC. In this study, we focused on the role of CASC11 as a ceRNA in CRC by regulating miR-646 and miR-381-3p targeting of RAB11FIP2.

**Methods:**

We identified the target microRNAs (miRNAs) of CASC11 and the target genes of miR-646 and miR-381-3p using bioinformatic methods. A dual-luciferase reporter assay was performed to validate the target relationship. Quantitative real-time PCR (qRT-PCR), western blotting (WB), and immunohistochemistry (IHC) were used to measure the RNA and protein expression levels. Rescue experiments *in vitro* and *in vivo* were performed to investigate the influence of the CASC11/miR-646 and miR-381-3p/RAB11FIP2 axis on CRC progression.

**Results:**

We found that CASC11 binds to miR-646 and miR-381-3p in the cytoplasm of CRC cells. Moreover, miR-646 and miR-381-3p inhibitors reversed the suppressive effect of CASC11 silencing on CRC growth and metastasis *in vitro* and *in vivo*. We further confirmed that RAB11FIP2 is a mutual target of miR-646 and miR-381-3p. The expression levels of CASC11 and RAB11FIP2 in CRC were positively correlated and reciprocally regulated. Further study showed that CASC11 played an important role in regulating PI3K/AKT pathway by miR-646 and miR-381-3p/RAB11FIP2 axis.

**Conclusion:**

Our study showed that CASC11 promotes the progression of CRC as a ceRNA by sponging miR-646 and miR-381-3p. Thus, CASC11 is a potential biomarker and a therapeutic target of CRC.

## Introduction

According to the World Cancer Report in 2018, colorectal cancer (CRC) is the third most frequent cancer and the second leading cause of cancer-related mortality (1). It is urgent to elucidate the critical molecular pathways involved in CRC and to develop effective therapeutic approaches for CRC. It has been established that non-coding genes occupy > 98% of the human genome, which far exceeds the proportion of coding genes (2, 3). Long non-coding RNAs (lncRNAs), RNA molecules longer than 200 nt in length have little or no protein-coding potential, and their functions are closely related to their subcellular localization (4, 5). In recent decades, an increasing number of lncRNAs have been found to be key regulators in tumour initiation and progression. Currently, the mechanisms of action of lncRNAs can be described by 4 modes: signal (functioning as indicators of transcriptional activity), decoy (acting as a “molecular sink” titrating away proteins and small regulatory RNAs), guide (directing the localization of ribonucleoprotein complex to specific targets), and scaffold (serving as central platforms upon which relevant molecular components are assembled) (6). Herein, lncRNAs acting as competitive endogenous RNAs (ceRNAs) of miRNAs are representative of the decoy mechanism (6). lncRNAs can crosstalk through their ability to compete for microRNA response elements (MREs), by which transcripts can actively communicate to each other to regulate their respective expression levels (7). The term “ceRNAs” refers to lncRNAs that share sequence identity and similarity with mRNA and can competitively bind to miRNAs to exert their function (7). Furthermore, extensive evidence has revealed that lncRNAs functioning as ceRNAs can bind to different miRNAs in different tumours. As reported, HOTAIR, a well-studied lncRNA, promotes non-small-cell lung cancer growth, invasion and metastasis by acting as a sponge of miR-149-5p (8) and facilitates CRC progression by sponging miR-214 (9).

We previously found that the lncRNA CASC11 plays an important role in the regulation of HNRNPK protein expression in CRC progression (10). CASC11 is located at the human chromosome 8q24 region, which is well known as a “genetic desert” because of the lack of any protein-coding oncogenes (10, 11). According to current studies, CASC11 can act as a critical tumour promoter in various tumours, such as gastric cancer (12), liver cancer (13) glioma (14) and CRC (10). The main functions of CASC11 are associated with binding to proteins or participating in ceRNA crosstalk. It has been reported that CASC11 acts as a ceRNA and promotes the proliferation and metastasis of gastric cancer by sponging miR-340-5p (12) Moreover, CASC11 facilitates the growth of glioma by adsorbing miR-498 (14). Given the versatile mechanisms of action of CASC11 in a variety of cancer types, we decided to explore the mechanisms of CASC11 as a ceRNA in CRC progression. To date, there has been no report regarding the ceRNA regulatory mechanism of CASC11 in CRC.

## Materials and Methods

### Cell Culture

FHC normal human colonic mucosal epithelial cells and SW480, LOVO, HCT116, RKO, Caco2, SW620, and LS174T human CRC cells were purchased from the American Type Culture Collection (ATCC, USA) and stored at 37°C in a 5% CO_2_ atmosphere. FHC cells were cultured in Dulbecco’s modified Eagle medium (DMEM, Gibco, USA) containing 20% foetal bovine serum (FBS, Gibco, USA), while the 7 CRC cell lines were grown in RPMI-1640 (Gibco, USA) supplemented with 10% FBS (Gibco, USA). We routinely tested for mycoplasma contamination by using the one-step Quickcolor Mycoplasma Detection Kit (Yise Medical Technology, Shanghai, China, #MD001), and all the cells used in our study were free of mycoplasma.

### Tissue Samples and Animals

CRC tissues and adjacent normal tissues were collected from surgical specimens of patients from 2016 to 2018. Female BALB/c nude mice approximately 5-6 weeks old were purchased from the Guangdong Animal Center (Guangzhou, China) and housed in an SPF-grade animal experimental centre at Southern Medical University.

### RNA Extraction and qRT-PCR

Total RNA was extracted with TRIzol (TaKaRa, Japan). RNA concentrations, and the 260/280 and 260/230 ratios were measured using a NanoDrop 2000 spectrophotometer (ThermoFisher, USA). Reverse transcription was performed using the PrimeScript RT reagent Kit (TaKaRa, Japan) and All-in-One miRNA qRT-PCR Kit (GeneCopoeia, USA). qRT-PCR was performed on an ABI 7500 Fluorescence Quantitative Detector with SYBR Green qPCR Mix (DBI Bioscience, Germany) and All-in-One miRNA qRT-PCR Kit (GeneCopoeia, USA). Fold changes=2^-ΔΔCt^ represent the relative ratios between the groups, with GAPDH or U6 serving as the internal standard. The sequences of the qRT-PCR primers were as follows: CASC11 Forward, 5’-ACCCTATGGAGAACCGAGAC-3’ and Reverse, 5’-GAGGACCAACTCAGTAGGAAAT-3’; RAB11FIP2 Forward, 5’-TGTCCGAGCAAGCCCAAAAG-3’ and Reverse, 5’- CTCCTTCCAAACTGGCTCAAG-3’; WEE1 Forward, 5’-AACAAGGATCTCCAGTCCACA-3’ and Reverse, 5’- GGGCAAGCGCAAAAATATCTG-3’; RAB30 Forward, 5’- TGCCTCGTCCGAAGATTCAC-3’ and Reverse, 5’- AGTAACTCTGGGTAATGGACCG-3’; GAPDH Forward, 5’-GGAGCGAGATCCCTCCAAAAT-3’ and Reverse, 5’- GGCTGTTGTCATACTTCTCATGG-3’; and U6 Forward, 5’-CTCGCTTCGGCAGCACA-3’ and Reverse, 5’-AACGCTTCACGAATTTGCGT-3’.

### Plasmid Construction and Transfection

The overexpression and interfering sequences of CASC11 reported previsouly (10) were used to construct the CASC11 overexpression plasmid and the intervention lentivirus (GeneChem, Shanghai, China). The miR-646 and miR-381-3p intervention viruses (LV-anti-miR-646 and LV-anti-miR-381-3p) were constructed by GeneChem (Shanghai, China). siRAB11FIP2 and its negative control siNC were purchased from RiboBio (Guangzhou, China); miRNA mimics and inhibitors were provided by GenePharma (Suzhou, China). Lipofectamine 3000 (Invitrogen, USA) was used for cell transfection according to the instructions of the manufacturer.

### CCK-8 Cell Proliferation Assay

A CCK-8 assay (KeyGen Biotech, Nanjing, China) was used to detect the cell proliferation rates. Briefly, the transfected cells were seeded into 96-well plates (1000 cells/well). The cell proliferation assay was measured every 24 hours for 6 consecutive days. The spectrophotometric absorbance of each sample was read at 450 nm. Each sample was assayed in 5 replicates and repeated 3 times independently.

### Plate Colony-Forming Assay

For colony formation assay, each well of a 6-well culture plate was seeded with 500 cells. After incubation at 37°C for 12 days, each well was washed three times with PBS, fixed with 4% paraformaldehyde for 30 min and stained with Giemsa solution for 10 min at room temperature. The number of colonies containing ≥ 50 cells was counted under a microscope and images were captured using a digital camera.

### Flow Cytometry Cell Cycle Analysis

After 48h, the transfected cells were digested by trypsinization and fixed in cold 75% ethanol at 4 °C overnight. Subsequently, the Cell Cycle Detection Kit (KeyGen Biotech, Nanjing, China) was used according to the manufacturer’s instruction. 1×10^6^ cells were incubated with 100 ul RNase A at 37°C for 30 min and stained with 400ul propidium iodide (PI) staining solution for 15 min at 4 °C in the dark. Then, a flow cytometric analysis was performed, and the percentage of cells in G0–G1, S, and G2–M phases were calculated using ModFit LT software (version 3.0, Verity, USA).

### Scratch Wound and Transwell Assays

The migratory capacity of cells was determined by scratch wound and Transwell assays. For the scratch wound assay, parallel lines were scratched on 6-well plates with 10-µl sterile tips when the transfected cells reached 90% confluence, followed by three washes with PBS to remove the cell debris. Images were then taken at 0, 24, and 48 h on an inverted microscope at 200× magnification. The scratch closure area was measured using ImageJ software (NIH, Bethesda, USA), and the equation for the healing rate was as follows: (% at 0 h) = (S0-ST)/S0 × 100%, where S0 and ST are the closure areas at 0 and T h, respectively. Next, 24-well plates with 8.0-μm pore membranes (Corning, NY, USA) were used for the Transwell assay. Transfected cells (1 × 10^5^/well) were seeded into the upper chamber containing serum-free RPMI-1640 media, while 600 µl media supplemented with 20% FBS was added to the lower chamber. Other experimental procedures were performed as described elsewhere (15).

### Western Blotting (WB)

Proteins were extracted with RIPA lysis buffer (KeyGen Biotech, Nanjing, China) and quantified by using a bicinchoninic acid protein quantification kit (KeyGen Biotech, Nanjing, China). Proteins were separated by SDS-PAGE and then transferred onto PVDF membranes (Millipore, Bedford, USA), followed by blocking with PBST solution containing 5% skim milk or 5% BSA for 1 h. Subsequently, the primary antibody was added, and the membranes were incubated at 4°C overnight, followed by a 1-h incubation with the corresponding secondary antibody at room temperature. Finally, Pierce ECL Western Blotting Substrate (FDbio Science, China) was used for the chemiluminescence assay. The following primary antibodies were used: anti-RAB11FIP2 (Abcam, UK, #ab180504), anti-AKT (Proteintech, USA, #10176–2-AP), anti-p-AKT (Ser473) (Proteintech, USA, #664441-Ig), anti-PI3K (Abcam, UK, #ab140307), anti-p-PI3K (Tyr607) (Abcam, UK, #ab182651) and anti-GAPDH (Proteintech, USA, #10494–1-AP). PI3K inhibitor LY294002 (Beyotime, Shanghai, China, #S1737) was used at a concentration (50 umol/L).

### Fluorescence *In Situ* Hybridization (FISH)

The Cy3-labelled lncRNA CASC11 probe was synthesized by RiboBio (Guangzhou, China). FITC-labelled miR-646 and miR-381-3p probes were purchased from GENESEED (Guangzhou, China). The subcellular localization of CASC11 was detected using a FISH kit (RiboBio, China) with U6 as the nuclear control and 18S as the cytoplasmic control. Finally, the colocalization of CASC11 and miR-646 or miR-381-3p in CRC cells was detected as previously described (16).

### Bioinformatics Predictions of miRNAs that Bind With CASC11 and Their Target Genes

The miRNA-binding sites of CASC11 were predicted by use of the bioinformatics databases LncTar (http://www.cuilab.cn/lnctar), LncBook (https://bigd.big.ac.cn/lncbook/index), RegRNA2.0 (http://regrna2.mbc.nctu.edu.tw/detection.html), and RNAhybrid (https://bibiserv.cebitec.uni-bielefeld.de/rnahybrid/). Filtering restrictions were as follows: total score, ≥140; normalized free energy, >1; and minimum free energy, < –20kcal/mol (17). The potential target genes of miR-646 and miR-381-3p were predicted by TargetScan Human (http://www.targetscan.org/vert_72/). In the results of Targetscan, miRNA binding sites of type “8mer” and “7mer-m8” were included, and the “context ++ score percentile” more than 80 was selected as the scoring cut-off (18).

### Dual-Luciferase Reporter Assay

Full-length wild-type (Wt) or mutant (Mut) CASC11 oligonucleotides were inserted into the pmirGLO vector, and the dual-luciferase reporter plasmids were synthesized by Ruibiotech (Beijing, China). The length of the RAB11FIP2 3’UTR was 4182 bp, and the Wt and Mut psiCHECK2‐RAB11FIP2-3’UTR vectors were constructed by IGEbio (Guangzhou, China) to contain regions > 200 bp in length both downstream and upstream of each binding site. CRC cells were co-transfected with miRNA mimics or miR-NC and the aforementioned dual-luciferase plasmids using Lipofectamine 3000. After 48 h of transfection, the cells were lysed, and the luciferase activities were determined using the Promega Dual-Luciferase Reporter Assay System in accordance with the manufacturer’s protocol.

### Immunohistochemistry (IHC)

IHC staining and scoring were performed as described elsewhere (19). The primary antibodies anti-RAB11FIP2 (Abcam, UK, #ab180504) and anti-Ki67 (Abcam, UK, #ab94276) were used.

### Immunofluorescence (IF)

The cells were fixed with 4% paraformaldehyde for 30 min and then treated with 0.5% Triton X-100 for 10 min, followed by 30 min of blocking with 1% BSA at room temperature. The primary antibodies anti-RAB11FIP2 (Abcam, UK, #ab180504), anti-AKT (Proteintech, USA, #10176–2-AP), and anti-p-AKT (Ser473) (Proteintech, USA, #664441-Ig) were added, and the wells were incubated overnight at 4°C. The next steps were the same as described previously (20).

### *In Vivo* Xenograft Experiments

Stably transfected SW480 cells were used for the construction of nude mouse models bearing subcutaneous tumours or splenic capsule-injected metastatic tumours. A total of 1 × 10^7^ SW480 cells were suspended in PBS and subcutaneously injected into the groin of nude mice (5-week-old female mice, n = 5 in each group). The tumour size was measured by a Vernier calliper from the 7th day of injection, and the tumour volume was calculated (V = length × width^2^/2). After 4 weeks, the tumour was removed and fixed with 10% neutral-buffered formalin, followed by haematoxylin and eosin (H&E) and IHC staining. SW480 cells (2 × 10^6^) were injected into the splenic capsule of nude mice (6-week-old female mice, n = 4 in each group) to generate hepatic metastases. The mice were sacrificed after 8 weeks, and their spleens and livers were removed and subjected to histopathological examination.

### Statistical Analyses

Statistical analyses were performed by using SPSS 21.0 (IBM, USA) and GraphPad Prism version 6.0 software (GraphPad Software, USA). Experimental data are represented as the mean ± SD. Intergroup differences were analysed by one-way analysis of variance (ANOVA) or the independent-samples *t*-*test*. Relationships between CASC11 or RAB11FIP2 expression and clinicopathologic parameters were determined by the *χ^2^* test. The linear correlation of gene expression was analysed with Spearman’s correlation coefficient. WB images were quantified by a Gel-Pro Analyzer (Media Cybernetics, USA). IHC images were processed with Image-Pro Plus 6.0 (Media Cybernetics, USA). *p* < 0.05 was considered significant: *, *p* < 0.05; **, *p* < 0.01; ***, *p* < 0.001.

## Experimental Results

### miR-646 and miR-381-3p Bind to CASC11 in the Cytoplasm

**Figure 1:**
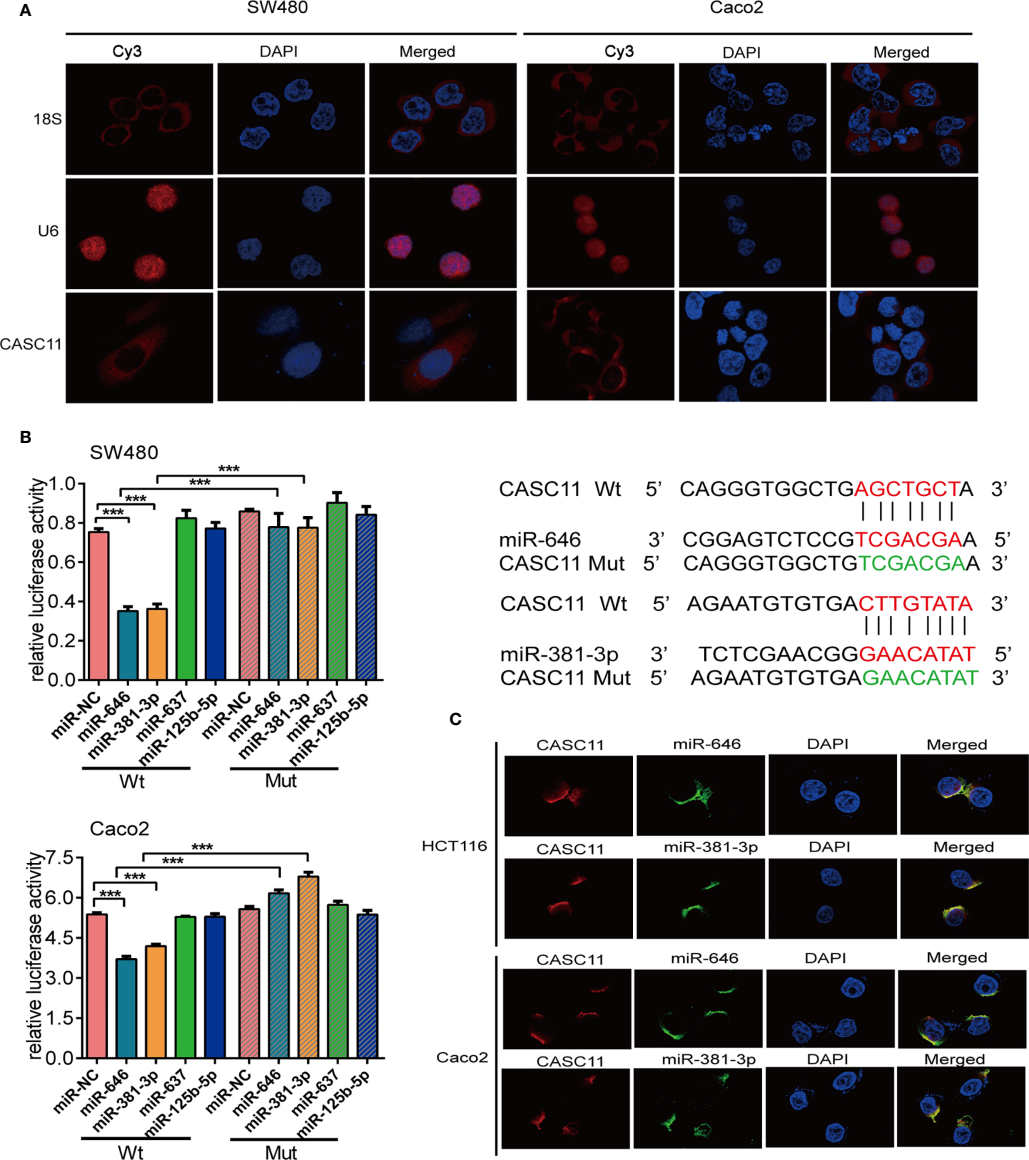
miR-646 and miR-381-3p bind to CASC11 in the cytoplasm. **(A)** Representative fluorescence images of the subcellular localization of CASC11. U6 and 18S were used as positive controls for the nucleus and cytoplasm, respectively. **(B)** The relative luciferase activity of SW480 and Caco2 cells was tested after co-transfection with wild-type or mutant CASC11 and miRNA mimics. Sequence alignment of miR-646 and miR-381-3p with the binding sites in the wild-type and mutant regions of CASC11 were shown on the right side. Error bars represent the means ± SD of 3 independent experiments. ***p <0.001. **(C)** FISH demonstrated that CASC11 colocalized with miR-646 and miR-381-3p in the cytoplasm of HCT116 and Caco2 cells. Red: CASC11 probes were labelled with Cy3. Green: miR-646 and miR-381-3p probes were labelled with FITC. Blue: nuclei were stained with 4’,6-diamidino-2-phenylindole (DAPI).

The subcellular localization of lncRNAs is closely related to their biological functions and molecular mechanisms (21). The endogenous expression of CASC11 was the highest in SW480 and the lowest in Caco2 cells among the 7 CRC cells, we chose these two cells for following experiment (**Additional File 1: Figure S1A**). We observed that CASC11 mainly resides in the cytoplasm of SW480 and Caco2 cells by performing FISH (**Figure 1A**). Then, we employed four bioinformatic databases to predict the target miRNAs of CASC11. The results showed that four candidate miRNAs, miR-646, miR-381-3p, miR-125b-5p, and miR-637, were predicted by more than three databases (**Additional File 1: Figure S1B**).

The binding relationship between CASC11 and miR-646, miR-381-3p, miR-125b-5p, or miR-637 was further validated by dual-luciferase reporter assays. The dual-luciferase reporter assay in SW480 and Caco2 cells showed that, compared with the those in control cells, the luciferase activities were reduced after co-transfection of miR-646 or miR-381-3p mimics and the CASC11-Wt plasmid, and there was no significant difference between the groups when miR-646 or miR-381-3p mimics and the CASC11-Mut plasmid were co-transfected. However, similar results were not obtained for miR-637 or miR-125b-5p (**Figure 1B**). In addition, RNA FISH revealed that CASC11 colocalized with miR-646 and miR-381-3p in the cytoplasm of HCT116 and Caco2 cells (**Figure 1C**). In summary, CASC11 binds to miR-646 and miR-381-3p in the cytoplasm of CRC cells.

### miR-646 and miR-381-3p Reverse CASC11-Mediated Phenotypes of CRC Cell Proliferation and Migration *In Vitro* and *In Vivo*

According to the endogenous expression levels of CASC11, miR-646, and miR-381-3p in 7 CRC cells, we selected different cell lines for transfection. CASC11 was knocked down in SW480 and SW620 cells *via* shCASC11 lentiviral infection, with the empty lentivirus used as control (shNC). The reduced expression of CASC11 was confirmed by qRT-PCR. Treatment with miR-646 and miR-381-3p inhibitors successfully decreased the expression levels of miR-646 and miR-381-3p in SW620 and HCT116 (**Additional File 1: Figure S1C**). qRT-PCR analysis also indicated that CASC11, miR-646, and miR-381-3p expression was significantly increased after transfecting CASC11 overexpression plasmids, miR-646 and miR-381-3p mimics, respectively (**Additional File 1: Figure S1D**).

**Figure 2:**
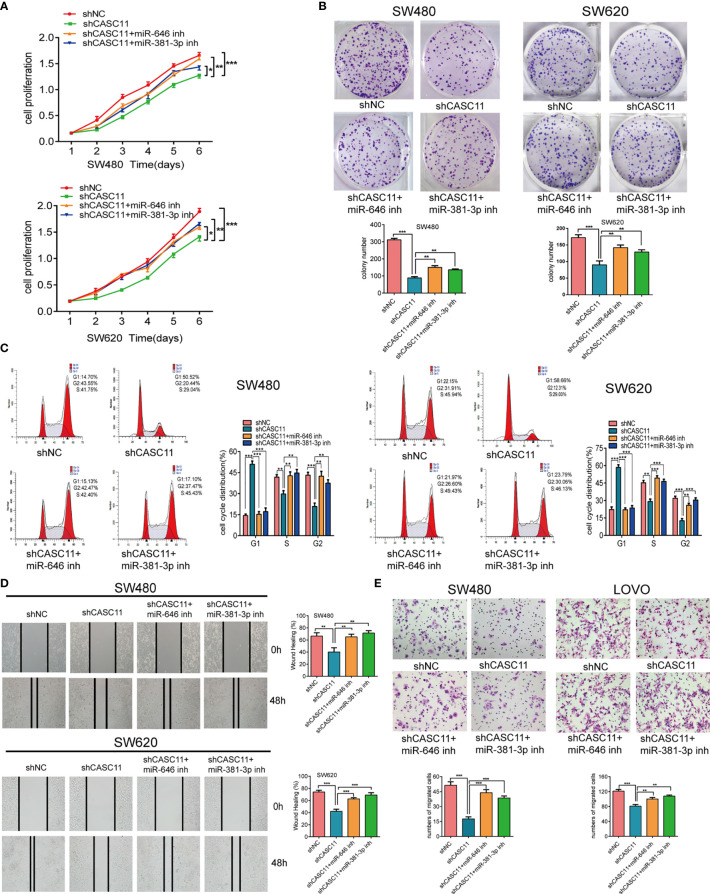
miR-646 and miR-381-3p inhibitors reverse CASC11 interference -mediated phenotypes of CRC cell proliferation and migration *in vitro*. **(A–C)** Cell proliferation was assessed by CCK-8 assay, colony formation assay, and flow cytometry cell cycle analysis in CASC11-knockdown SW480 and SW620 cells. Error bars represent the means ± SD of 3 independent experiments. **(D)** Cell migration was analysed by scratch wound assay in CASC11-knockdown cells. The bar chart represents the percentage of distance at 48 h divided by the distance at 0 h. Error bars represent the means ± SD of 3 independent experiments. **(E)** Transwell migration assay used to evaluate the migration ability of cells with downregulated CASC11. Error bars represent the means ± SD of 5 different fields. *p < 0.05; **p < 0.01; ***p < 0.001.

To confirm the effects of miR-646 and miR-381-3p on CASC11-mediated proliferation and migration of CRC cells, we conducted a series of *in vitro* functional assays. Both the CCK-8 assay and the plate colony-forming assay indicated that CASC11 interference restricted the growth of SW480 and SW620 cells, while this effect was reversed by the miR-646 and miR-381-3p inhibitors (**Figures 2A, B**). Flow cytometry cell cycle analysis revealed that CASC11 interference induced G1 phase arrest in SW480 and SW620 cells, which in turn inhibited cell growth; this effect was also reversed by the miR-646 and miR-381-3p inhibitors, which increased the ratio of cells at G2 phase and accelerated cell growth (**Figure 2C**). Both the scratch wound assay and the Transwell migration assay confirmed that CASC11 interference suppressed the migration of CRC cells, while this effect was reversed by the miR-646 and miR-381-3p inhibitors, which in turn enhanced cell migration (**Figures 2D, E**). In addition, functional assays in RKO and SW620 cells confirmed that miR-646 and miR-381-3p mimics could cancel the promoting effects of CASC11 overexpression on cell proliferation and migration (**Additional File 2: Figure S2A–D**). The abovementioned results confirmed in different ways that miR-646 and miR-381-3p can reverse CASC11-mediated proliferation and migration of CRC cells *in vitro*.

**Figure 3:**
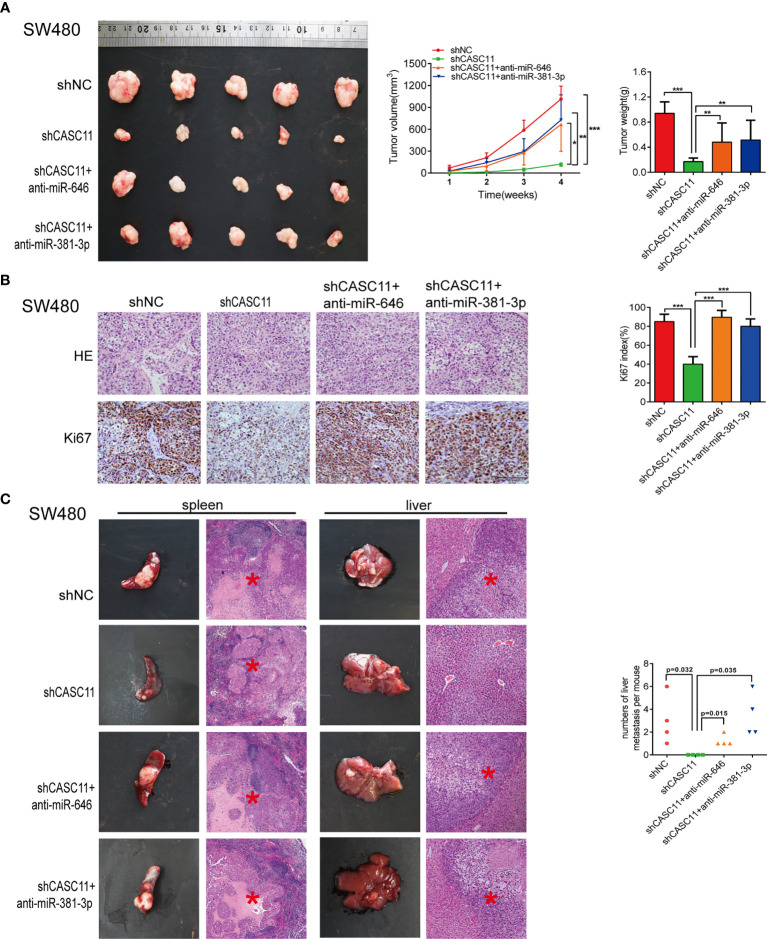
miR-646 and miR-381-3p inhibitors reverse CASC11 interference -mediated phenotypes of CRC cell proliferation and migration *in vivo*. **(A)** Mouse models of subcutaneous tumours developed from CASC11-knockdown SW480 cells, which were treated with anti-miR-646 or anti-miR-381-3p (n=5). Left: Images of the tumour mass of each group at the endpoint of the experiment. Middle: tumour growth curves. The data were calculated as the mean tumour volumes ± SD for 5 samples. Right: Tumour weights of the xenografts upon euthanasia at day 28. **(B)** Representative images of Ki67-positive sections of subcutaneous tumours by IHC assay. The error bars in all graphs represent the means ± SD of 3 different fields. **(C)** Representative images of splenic tumours, hepatic metastases, and haematoxylin and eosin (H&E) staining. The numbers of hepatic metastases per mouse are indicated on the right side (n = 4). *p < 0.05; **p < 0.01; ***p < 0.001.

To further demonstrate the role of miR-646 and miR-381-3p in CASC11-mediated proliferation and migration of CRC cells *in vivo*, we established SW480 nude mouse models bearing subcutaneous tumours or splenic capsule-injected liver metastatic tumours. SW480 cells transfected with shNC, shCASC11, shCASC11+LV-anti-miR-646, or shCASC11+LV-anti-miR-381-3p were injected. In the subcutaneous tumour mouse model, there was a notable reduction in the tumour growth rate in the shCASC11 group compared with the shNC group, while this growth reduction was reversed by LV-anti-miR-646 and LV-anti-miR-381-3p. After sacrificing the mice, we noted that LV-anti-miR-646 and LV-anti-miR-381-3p could partly counterbalance the effect of CASC11 knockdown on reducing the tumour volume and weight (**Figure 3A**). IHC confirmed that the Ki67 index was downregulated by shCASC11 and restored by LV-anti-miR-646 and LV-anti-miR-381-3p (**Figure 3B**). In the hepatic metastasis model, the average number of metastatic nodules in the shCASC11 group (0) was obviously reduced compared with that in the shNC group (3.0 ± 2.16), whereas suppressing the expression of miR-646 (1.25 ± 0.5) and miR-381-3p (3.5 ± 1.91) increased the number of metastatic nodules (**Figure 3C**). There were no metastatic nodules in any other organs in any group. In summary, we found that in nude mouse models of subcutaneous and splenic capsule-injected liver metastatic tumours, LV-anti-miR-646 and LV-anti-miR-381-3p could reverse the inhibitory effects of CASC11 knockdown on SW480 cells *in vivo*.

### RAB11FIP2 Is a Mutual Target of miR-646 and miR-381-3p

**Figure 4:**
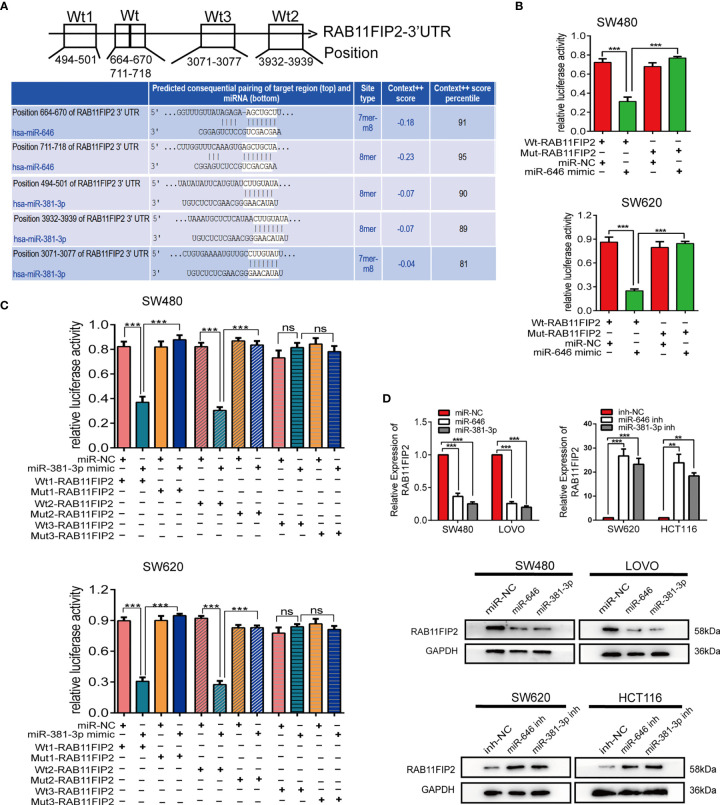
miR-646 and miR-381-3p co-target and negatively regulate RAB11FIP2. **(A)** Predicted binding sites of miR-646 and miR-381-3p on the RAB11FIP2 3’UTR sequence. The white nucleotides are the seed sequences of miR-646 and miR-381-3p. **(B, C)** Luciferase activities were measured in CRC cells co-transfected with a luciferase reporter containing the wild-type RAB11FIP2 3’UTR and miR-646 and miR-381-3p mimics or the mutant RAB11FIP2 3’UTR. Data are presented as the relative ratio of Renilla luciferase activity and firefly luciferase activity. **(D)** The relative expression levels of RAB11FIP2 in CRC cells transfected with miR-646 and miR-381-3p mimics or inhibitors were determined by qRT-PCR and WB. Error bars represent the means ± SD of 3 independent experiments. ns, no significance; **p < 0.01; ***p <0.001.

The target genes of miR-646 and miR-381-3p were predicted by TargetScan. Venn diagrams showed that there were 9 mutual target genes of miR-646 and miR-381-3p (**Additional File 3: Figure S3A**). Among them, *RAB11FIP2, RAB30*, and *WEE1* were selected for further validation because they have been reported to play a key role in tumour development. qRT-PCR assays were performed on 8 different cell lines, and the expression levels of the above three target genes, miR-646, and miR-381-3p were determined. Correlation analyses revealed a clear negative correlation between RAB11FIP2 and miR-646 (*r* = -0.9696, *p* = 0.0003) or miR-381-3p (*r* = -0.7684, *p* = 0.036), whereas RAB30 (*r* = -0.2625, *p* > 0.05; *r* = 0.2328, *p* > 0.05) and WEE1 (*r* = -0.0260, *p* > 0.05; *r* = -0.3473, *p* > 0.05) were not inversely correlated with miR-646 or miR-381-3p in these cells (**Additional File 3: Figure S3B**). Hence, we chose *RAB11FIP2* as the mutual target gene for further validation. TargetScan predicted two high-scoring binding sites for miR-646 in the RAB11FIP2 3’UTR and three for miR-381-3p (**Figure 4A**). Luciferase reporter assays revealed that the overexpression of miR-646 obviously decreased the luciferase activity of RAB11FIP2-3’UTR-Wt but not RAB11FIP2-3’UTR-Mut in SW480 and SW620 cells (**Figure 4B**). Compared with miR-NC, miR-381-3p mimics significantly decreased the luciferase activity of RAB11FIP2-3’UTR-Wt1 and -Wt2 but not RAB11FIP2-3’UTR-Mut1 and -Mut2. The luciferase activity of RAB11FIP2-3’UTR-Wt3 was not significantly altered by miR-381-3p mimics compared to miR-NC (**Figure 4C**). Accordingly, it can be concluded that miR-646 and miR-381-3p can bind to the RAB11FIP2 3’UTR.

Considering that the binding of a miRNA to its target gene is followed by mRNA degradation or translational inhibition, qRT-PCR and WB were employed to probe the regulatory relationship between miR-646 or miR-381-3p and RAB11FIP2. Compared with the control transfection, transfection with miR-646 or miR-381-3p mimics led to a decrease in the mRNA and protein levels of RAB11FIP2, whereas transfection with the miR-646 or miR-381-3p inhibitor led to an increase in the mRNA and protein levels of RAB11FIP2 (**Figure 4D**). Therefore, it can be concluded that miR-646 and miR-381-3p negatively regulate RAB11FIP2. In summary, miR-646 and miR-381-3p bind to the RAB11FIP2 3’UTR and inhibit the translation of RAB11FIP2.

### RAB11FIP2 and CASC11 Are Reciprocally Regulated by Sponging miR-646 and miR-381-3p

**Figure 5:**
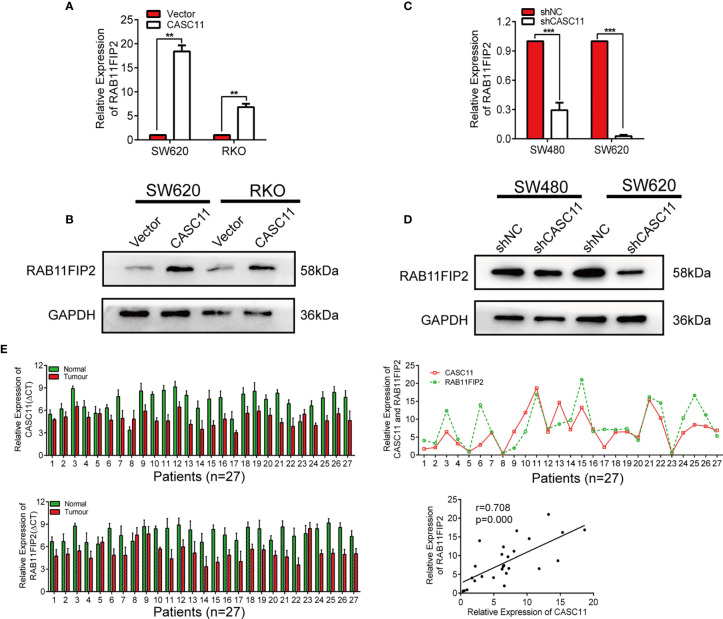
CASC11 positively regulated RAB11FIP2 expression in CRC cells. **(A-D)** The relative expression levels of RAB11FIP2 in CRC cells transfected with CASC11 overexpression or interference reagents were determined by qRT-PCR and WB. **(E)** Expression of CASC11 and RAB11FIP2 in CRC tissues and the relationship between them (n=27). Left panel, qRT-PCR analysis of CASC11 and RAB11FIP2 expression in 27 paired human CRC tissues and adjacent normal tissues. Right panel, spearman correlation analysis showed a positive relationship between CASC11 expression levels and RAB11FIP2 mRNA levels in 27 CRC tissue samples. Error bars represent the means ± SD of 3 independent experiments. **p < 0.01; ***p <0.001.

To further confirm whether CASC11 regulates the miR-646- and miR-381-3p-targeted gene RAB11FIP2, we increased CASC11 expression in CRC cells and found that it resulted in elevated expression of RAB11FIP2 at both the mRNA and protein levels (**Figures 5A, B**). In contrast, silencing CASC11 expression caused a decrease in RAB11FIP2 expression (**Figures 5C, D**). We also determined the expression of CASC11 and RAB11FIP2 in 27 pairs of CRC tissues by qRT-PCR. Our results revealed that the expression levels of both were higher in cancerous tissues than in normal adjacent tissues, indicating clear positive correlations (**Figure 5E**). Furthermore, to investigate the clinicopathologic significance of CASC11 and RAB11FIP2, the expression levels of CASC11 or RAB11FIP2 in 27 pairs of CRC tissues by qRT-PCR were divided into a high expression group and a low expression group by the median CASC11 or RAB11FIP2 expression. As summarized in Table S1 (**Additional File 6: Table S1**), the high expression level of CASC11 was significantly correlated with tumour size, lymph-vascular invasion, lymph metastasis, and T-stage. RAB11FIP2 expression was positively associated with tumour size and T-stage. We further evaluated the expression levels of miR-646 and miR-381-3p in 27 pairs of CRC tissues. The expression of miR-646 was lower in 23 of 27 CRC specimens compared to the adjacent normal tissues by qRT-PCR (*p <*0.001), as well as miR-381-3p (*p <*0.01) (**Additional File 5: Figure S5A**).

The successful construction of siRAB11FIP2-SW480 and siRAB11FIP2-SW620 cells was confirmed by qRT-PCR and WB (**Additional File 4: Figure S4A, B**). RAB11FIP2 interference led to a decrease in the expression of CASC11 compared to siNC (**Additional File 4: Figure S4C**). In addition, the WB results revealed that the miR-646 and miR-381-3p inhibitors could restore the decrease in RAB11FIP2 protein levels caused by CASC11 knockdown (**Additional File 4: Figure S4D**). The detection of 67 pairs of CRC tissues by IHC indicated that the positive rates of the RAB11FIP2 protein were significantly higher in the cancerous tissues than in the para-carcinoma tissues (**Additional File 4: Figure S4E**). The *in vitro* proliferation and migration assays in LOVO and RKO cells confirmed that siRAB11FIP2 could reverse the effect on the proliferation and migration of CRC cells caused by CASC11 overexpression (**Additional File 4: Figure S4F–H**). In summary, CASC11 and RAB11FIP2 are positively regulated by each other by sponging miR-646 and miR-381-3p.

### CASC11 Is a PI3K/AKT Signaling Pathway Regulator in CRC Cells

**Figure 6:**
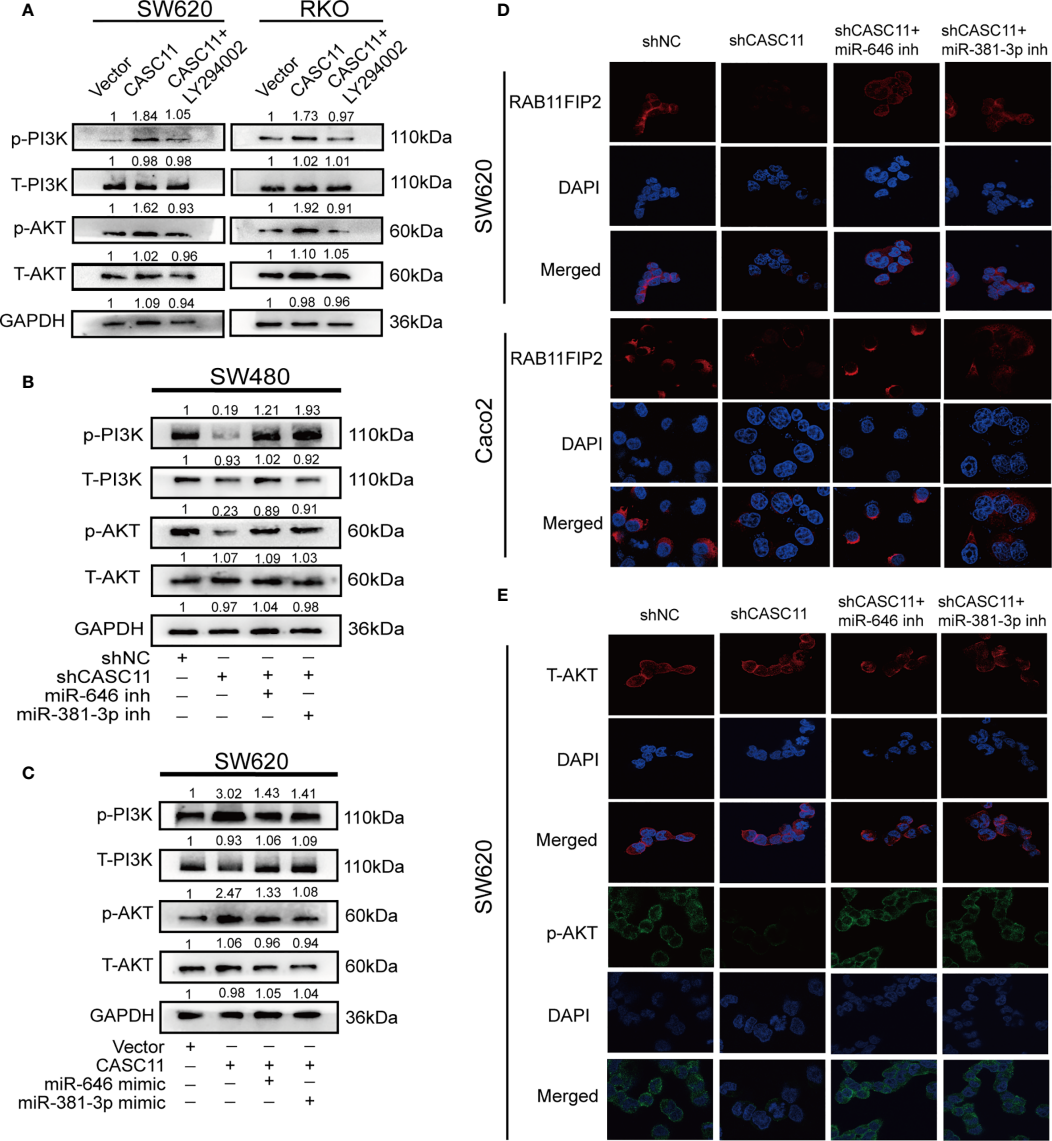
CASC11 is a PI3K/AKT signalling pathway regulator in CRC cells. **(A)** The levels of the main molecules of the PI3K/AKT pathway in CRC cells transfected with CASC11 overexpression, followed by treatment with LY294002. **(B)** The main molecules of the PI3K/AKT pathway in CRC cells co-transfected with shCASC11 and the miR-646 or miR-381-3p inhibitor were analyzed. **(C)** The main molecules of the PI3K/AKT pathway in CRC cells co-transfected with CASC11 overexpression and miR-646 or miR-381-3p mimics were shown. **(D, E)** IF microscopy of the localization and expression of RAB11FIP2, AKT and p-AKT in CASC11-knockdown cells co-transfected with the miR-646 or miR-381-3p inhibitor.

According to a previous study, CASC11 can promote the progression and metastasis of liver cancer by activating the PI3K/AKT pathway (13), and RAB11FIP2 could facilitate the metastasis and progression of CRC through the same pathway (22). For this reason, we wondered whether CASC11 involved in the growth and metastasis of CRC through the PI3K/AKT pathway. It was thus determined that, compared to the control vector, the CASC11 overexpression group promoted the phosphorylation of PI3K and AKT (p-PI3K and p-AKT), while treatment with the PI3K inhibitor LY294002 restrained the above effect of CASC11 overexpression (**Figure 6A**). CASC11 knockdown reduced p-PI3K and p-AKT, which could be restored by the miR-646 and miR-381-3p inhibitors (**Figure 6B**). Moreover, miR-646 and miR-381-3p mimics partly reversed the increase in p-PI3K and p-AKT caused by CASC11 overexpression (**Figure 6C**). No significant change was noted in total PI3K and AKT (T-PI3K and T-AKT) under any condition. IF revealed that CASC11 interference reduced RAB11FIP2 and p-AKT levels compared with the control treatment, and this effect could be restored by the addition of the miR-646 and miR-381-3p inhibitors (**Figures 6D, E**). In summary, these data suggested that CASC11 played an important role in regulating PI3K/AKT pathway by miR-646 and miR-381-3p/RAB11FIP2 axis.

## Discussion

In this study, we found that miR-646 and miR-381-3p bind not only to CASC11 but also to the RAB11FIP2 3’UTR. The expression levels of CASC11 and RAB11FIP2 in CRC tissues were found to be higher than those in para-cancerous tissues and were positively correlated. miR-646 and miR-381-3p reversed CASC11-mediated phenotypes of CRC progression and restored the regulatory effects of CASC11 on RAB11FIP2 and the phosphorylation levels of the PI3K/AKT pathway.

Several past studies have reported that, as an oncogene, CASC11 participates in various biological processes of malignant tumours. For instance, CASC11 is highly expressed in gastric cancer tissues and is involved in the promotion of the growth, invasion, and metastasis of gastric cancer *via* the CASC11/miR-340-5p/CDK1 axis (12). In addition, CASC11 is highly expressed in liver cancer cells and promotes liver cancer progression by activating the PI3K/AKT pathway (13). Moreover, CASC11 targets the miR-498/FOXO3 axis and accelerates the proliferation and cell cycle progression of non-small-cell lung cancer (23). However, CASC11 is rarely reported in relation to CRC, with the only study reported by our group. We previously reported that CASC11 promotes CRC progression by binding to the HNRNPK protein and activating the Wnt/β-catenin pathway (10). The present study first discovered that CASC11 acts as a ceRNA and competes with RAB11FIP2 to bind to miR-646 and miR-381-3p and ultimately modulate CRC proliferation and metastasis.

ceRNAs refer to coding or non-coding RNAs that share sequence identity or similarity with mRNAs and competitively bind to miRNAs (7). The ceRNA mechanism was first reported by Poliseno et al., who showed that the pseudogene *PTENP1* is not translated into protein but rather acts as a molecular decoy of PTEN (24). LncRNAs are an important class of non-coding RNAs that function as ceRNAs. Linc-MD1 sponges miR-133 and miR-135 to regulate the transcription factors MAML1 and MEF2C, thus activating the expression of muscle differentiation-specific genes (25). LncRNA-KRTAP5-AS1 and lncRNA-TUBB2A can function as ceRNAs that bind miR-596 and miR-3620-3p and regulate Claudin-4 to promote the growth, metastasis, and epithelial-mesenchymal transition (EMT) of gastric cancer (26).

When acting as a ceRNA, one lncRNA molecule can bind with multiple miRNAs, and one miRNA can be targeted by multiple lncRNAs. miRNAs can be classified into cancer-promoting miRNAs, cancer-suppressing miRNAs, and dual-effect miRNAs (27, 28). This study confirmed that both miR-646 and miR-381-3p can site-specifically bind to CASC11. In addition, miR-646 and miR-381-3p are derived from human chromosomes 20 and 14, respectively, and their roles in tumours have been extensively reported. miR-646 has been indicated to function as a tumour suppressor in retinoblastoma (29), endometrial carcinoma (30), non-small-cell lung cancer (31), laryngeal squamous cell carcinoma (32), and CRC (33). miR-381-3p also functions as a tumour suppressor in non-small-cell lung cancer (34), oral squamous cell carcinoma (35), breast cancer (36), and cervical cancer (37). However, Zhao et al. (38) demonstrated that miR-381-3p can promote renal cell carcinoma by inhibiting apoptosis and necrosis. However, miR-381-3p has not been reported in CRC-related research. The present study revealed that miR-646 and miR-381-3p were significantly downregulated in CRC tissues and cells; subsequent functional assays *in vitro* and *in vivo* ascertained that the tumour-promoting effect of CASC11 could be cancelled by miR-646 and miR-381-3p in CRC; thus, miR-646 and miR-381-3p may function as tumour suppressors in CRC.

RAB11FIP2 is a member of the RAB11 family interacting proteins (RAB11-FIP) that plays crucial roles in tumour growth and metastasis (39). For example, Gidon et al. found that RAB11FIP2, acting as an element of the membrane platform, regulated the plasma membrane recycling of melanoma cells (40). It was reported that RAB11FIP2 was upregulated in gastric cancer tissues and that the overexpression of RAB11FIP2 facilitated the metastasis of gastric cancer (41). Dong et al. discovered that the overexpression of RAB11FIP2 may elevate the secretion of PAI-1, which results in the promotion of the proliferation, angiogenesis, and migration of CRC cells (42). Similarly, our study showed that RAB11FIP2, which is a mutual target of miR-646 and miR-381-3p, was highly expressed in CRC tissues and cells. CASC11 and RAB11FIP2 were positively regulated by each other. However, how RAB11FIP2 was causing an increase in CASC11 expression? After searching the relevant database, we found that there was no evidence that RAB11FIP2 could act as a transcription factor binding directly to CASC11 promoter. As RAB11FIP2 protein containing C2 domain and FIP domain has no DNA binding domain, it is impossible to bind directly to CASC11 promoter. In our work, RAB11FIP2 regulated PI3K/AKT signalling pathway. Our previous study found c-Myc directly bound to the promoter regions of CASC11 (10). Furthermore, it is widely accepted that the aberrant activation of PI3K/AKT signalling elevates c-Myc expression (43, 44). We speculated that RAB11FIP2 caused an increase in CASC11 expression by regulating PI3K/AKT/c-Myc signalling.

**Figure 7:**
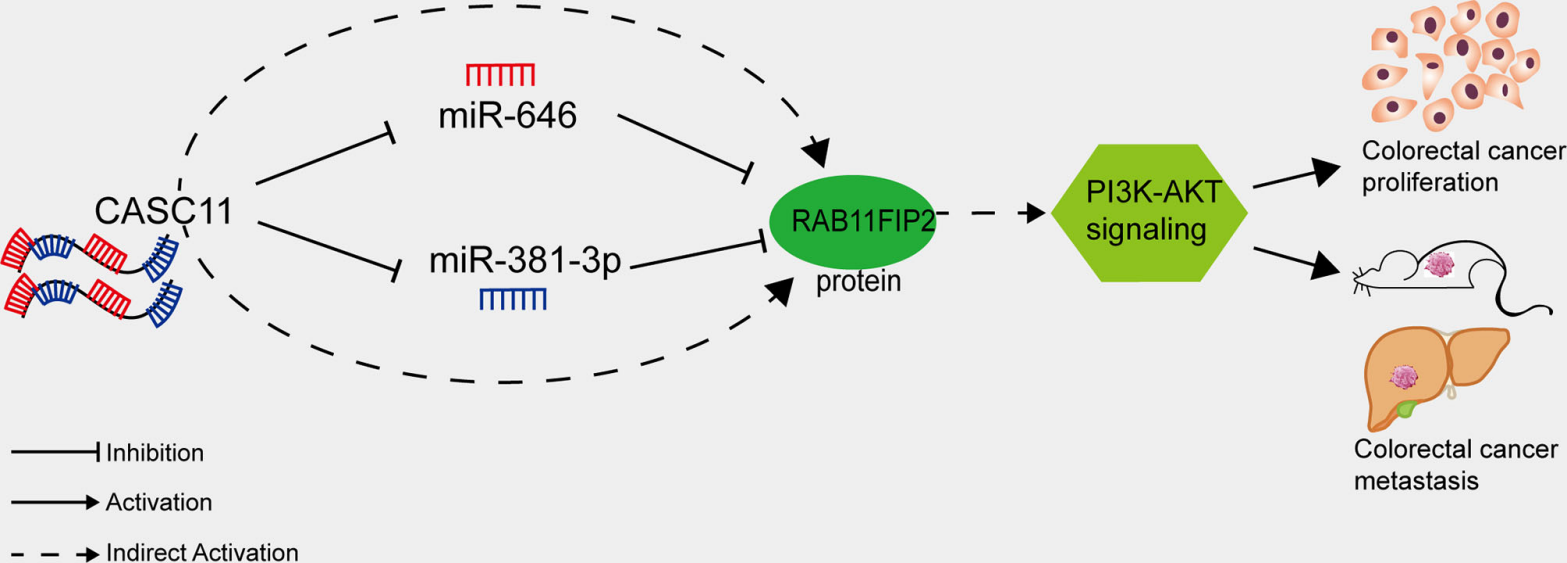
Proposed model of how CASC11 regulates CRC proliferation and metastasis through the miR-646 and miR-381-3p/RAB11FIP2 axis.

The PI3K/AKT pathway regulates cell proliferation, differentiation, apoptosis, and angiogenesis in CRC and various other tumour types (45). Han et al. reported that CASC11 can bind EZH2 and mediate PTEN silencing, which activates the PI3K/AKT pathway and promotes the progression of liver cancer (13). Xu et al. showed that RAB11FIP2 promotes CRC progression by upregulating MMP7 and activating the PI3K/AKT pathway (22). It could be inferred that CASC11 may play an important role in regulating PI3K/AKT pathway in CRC cells. We demonstrated in this study that interfering with CASC11 could reduce p-PI3K and p-AKT expression, which could be restored by the miR-646 and miR-381-3p inhibitors. Conversely, CASC11 overexpression increased p-PI3K and p-AKT levels, which could be restrained by miR-646 and miR-381-3p mimics. Our data thus suggest that CASC11 played an important role in regulating PI3K/AKT pathway by miR-646 and miR-381-3p/RAB11FIP2 axis in CRC cells (**Figure 7**).

## Conclusion

In summary, in CRC, the lncRNA CASC11 acts not only as a tumour promoter that binds to related proteins and activates the Wnt/β-catenin pathway but also as a sponge of miR-646 and miR-381-3p to upregulate RAB11FIP2 and regulate the PI3K/AKT pathway, thus promoting CRC progression. Our previous and current research suggests that CASC11 is a potential biomarker and a promising therapeutic target of CRC. However, while our findings link CASC11 and RAB11FIP2 to the PI3K/AKT pathway, their mechanism in activating PI3K/AKT signalling needs to be further validated in depth.

## Data Availability Statement

The original contributions presented in the study are included in the article/**Supplementary Material**. Further inquiries can be directed to the corresponding author.

## Ethics Statement

The studies involving human participants were reviewed and approved by the Ethics Agreement of Nanfang Hospital of Southern Medical University. The patients/participants provided their written informed consent to participate in this study. The animal study was reviewed and approved by the animal ethics committee of Southern Medical University. Written informed consent was obtained from the individual(s) for the publication of any potentially identifiable images or data included in this article.

## Author Contributions

XnL contributed to study design, obtaining funding, and study supervision. WZ carried out the experiments and wrote the manuscript. XmL contributed in data acquisition and statistics analysis. WjZ and YxL interpreted the results. WhL contributed in the collection of patient samples and data input. All authors contributed to the article and approved the submitted version.

## Funding

This study was supported by the National Natural Science Foundation of China (grant numbers 81874074, 81672429, and 82072705).

## Conflict of Interest

The authors declare that the research was conducted in the absence of any commercial or financial relationships that could be construed as a potential conflict of interest.

## Supplementary Material

Supplementary Figure 1 **(A)** qRT-PCR assay showed the expression of CASC11 in 8 cell lines. **(B)** Venn diagrams showing the intersection set of CASC11-binding miRNAs. **(C)** CASC11 in SW480 and SW620 cells after knockdown of CASC11 were detected by RT-qPCR. Treatment with miR-646 and miR-381-3p inhibitor successfully decreased the expression levels of miR-646 and miR-381-3p in SW620 and HCT116. **(D)** The overexpression of CASC11 in SW620 and RKO cells, miR-646 and miR-381-3p in SW480 and LOVO cells were confirmed by qRT-PCR. Error bars indicate the means ± SD of 3 independent experiments. **p* < 0.05; ***p* < 0.01; ****p* < 0.001.

Supplementary Figure 2 miR-646 and miR-381-3p mimics reverse CASC11 overexpression-mediated phenotypes of CRC cell proliferation and migration *in vitro*. **(A, B)** Cell proliferation was assessed by CCK-8 assay and flow cytometry cell-cycle analysis in CASC11 overexpression RKO and SW620 cells. Error bars represent the means ± SD of 3 independent experiments. **(C, D)** Cell migration was analyzed by scratch wound assay and Transwell migration assay in CASC11 overexpression RKO and SW620 cells. Error bars represent the means ± SD of 3 independent experiments or 5 different fields, respectively. **p* < 0.05; ***p* < 0.01; ****p* < 0.001.

Supplementary Figure 3 Selecting RAB11FIP2 as a mutual target gene of miR-646 and miR-381-3p. **(A)** Venn diagrams showing the intersection set of mutual target genes of miR-646 and miR-381-3p. **(B)** qRT-PCR assays showed expressions of RAB11FIP2, WEE1, RAB30, miR-646, and miR-381-3p in 8 cell lines. Spearman correlation analysis showed a negative relationship between RAB11FIP2 mRNA and the above two miRNAs. Error bars indicate the means ± SD of 3 independent experiments. **p* < 0.05; ***p* < 0.01; ****p* < 0.001.

Supplementary Figure 4 RAB11FIP2 positively regulated CASC11 expression in CRC cells, and RAB11FIP2 knockdown can prohibit CRC cell proliferation and migration mediated by CASC11 overexpression. **(A, B)** The successful construction of siRAB11FIP2 cells was confirmed by qRT-PCR and WB. **(C)** The relative expressions of CASC11 in CRC cells transfected with siRAB11FIP2 by qRT-PCR. **(D)** miR-646 and miR-381-3p inhibitors rescued a decrease in RAB11FIP2 protein levels caused by CASC11 knockdown.**(E)** Immunohistochemical staining (IHC) evaluation of the expression of RAB11FIP2 in paraffin-embedded human CRC tissues and adjacent normal tissues (n=67). The error bars in all graphs represent the means ± SD of 3 different fields. **(F–H)** siRAB11FIP2 reverses CASC11 overexpression-mediated phenotypes of CRC cell proliferation and migration *in vitro*. Cell proliferation was assessed by CCK-8 assay, and cell migration was analyzed by scratch wound assay and Transwell migration assay. **p* < 0.05; ***p* < 0.01; ****p* < 0.001.

Supplementary Figure 5 **(A)** qRT-PCR analysis of miR-646 and miR-381-3p expression in 27 paired CRC tissues. Left panel, the results were presented as the fold change in tumour tissues relative to the matched adjacent normal tissues. Right panel, comparison of miR-646 and miR-381-3p expression in 27 paired CRC tissues (T) and matched normal tissues (N). Error bars indicate the means ± SD of 3 independent experiments.

## Abbreviations

CRC, Colorectal cancer; CASC11, Cancer susceptibility candidate 11; BSA, Albumin from bovine serum; qRT-PCR, Quantitative real-time PCR; WB, Western blotting; FISH, Fluorescence *in situ* hybridization; CCK-8, Cell counting kit-8; FBS, Fetal bovine serum; IF, Immunofluorescence; IHC, Immunohistochemistry; RAB11FIP2, RAB11 family-interacting protein 2; NC, Negative control.
