# Supplementary figures and images for: The LncRNA CASC11 Promotes Colorectal Cancer Cell Proliferation and Migration by Adsorbing miR-646 and miR-381-3p to Upregulate Their Target RAB11FIP2
Source: Front Oncol. 2021 Apr 15;11:657650. doi: 10.3389/fonc.2021.657650 (PMC8084185; doi:10.3389/fonc.2021.657650)

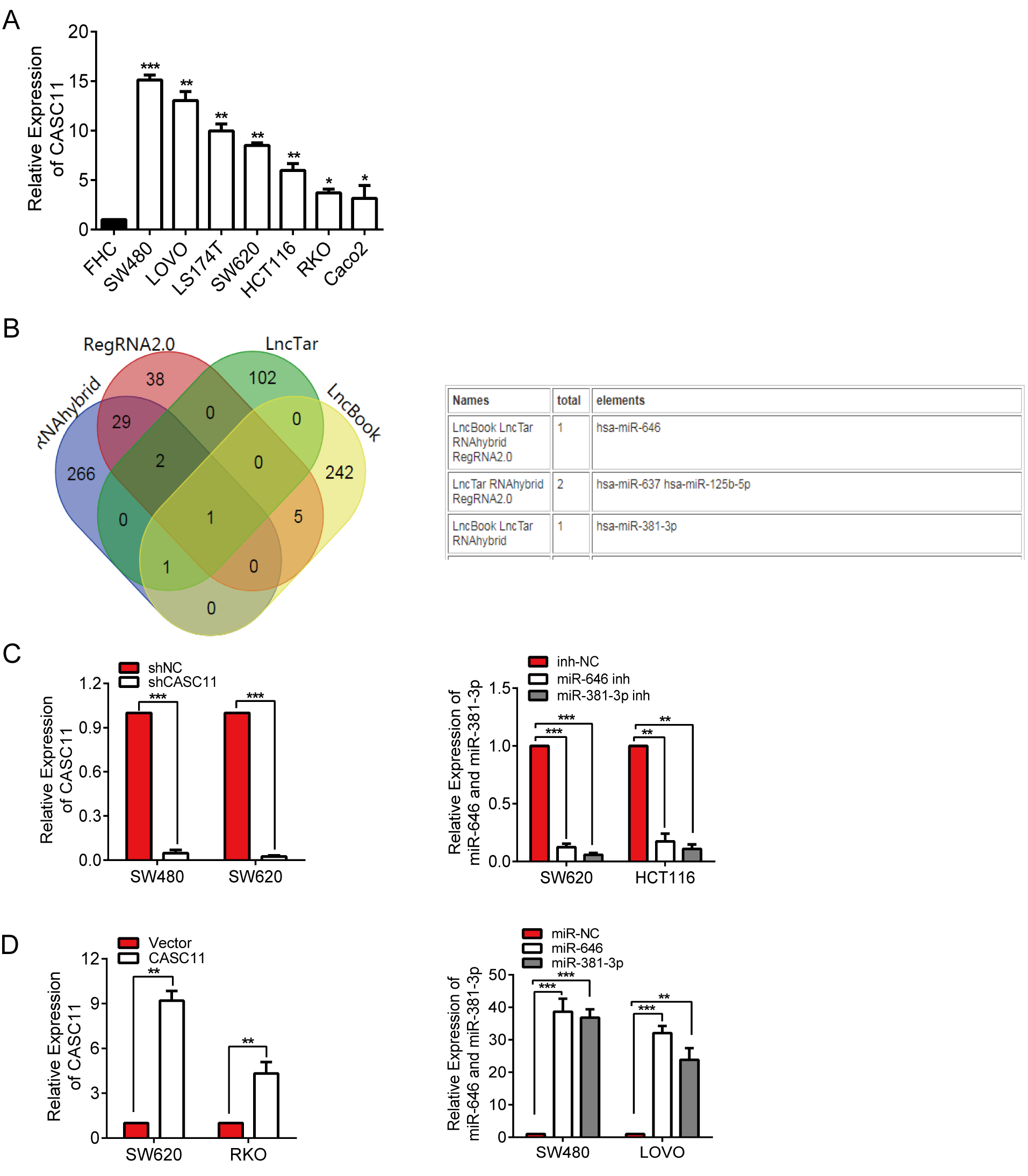

Supplement: Supplementary Figure 1 — (A) qRT-PCR assay showed the expression of CASC11 in 8 cell lines. (B) Venn diagrams showing the intersection set of CASC11-binding miRNAs. (C) CASC11 in SW480 and SW620 cells after knockdown of CASC11 were detected by RT-qPCR. Treatment with miR-646 and miR-381-3p inhibitor successfully decreased the expression levels of miR-646 and miR-381-3p in SW620 and HCT116. (D) The overexpression of CASC11 in SW620 and RKO cells, miR-646 and miR-381-3p in SW480 and LOVO cells were confirmed by qRT-PCR. Error bars indicate the means ± SD of 3 independent experiments. *p < 0.05; **p < 0.01; ***p < 0.001. [file Image_1.tif]

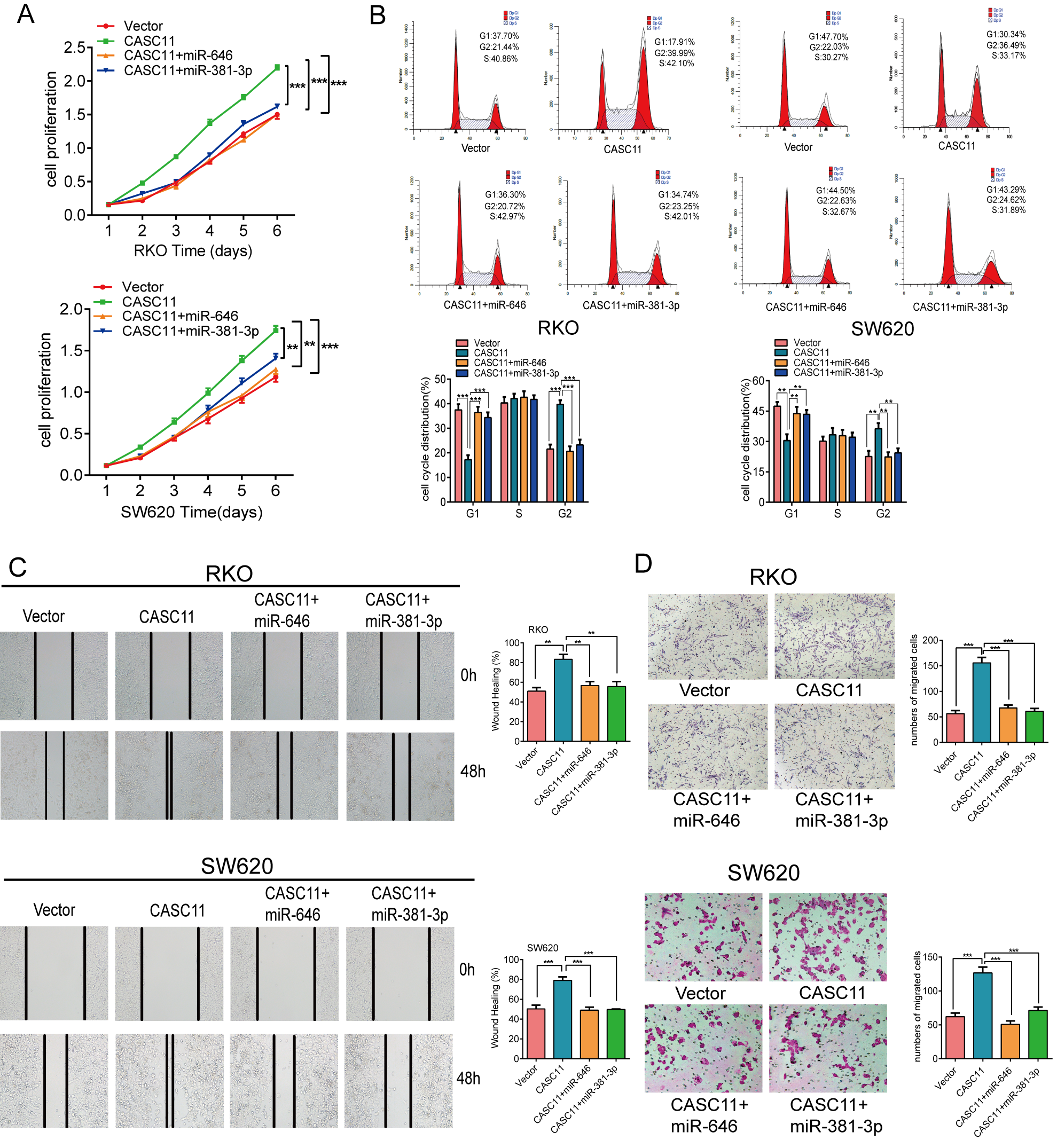

Supplement: Supplementary Figure 2 — miR-646 and miR-381-3p mimics reverse CASC11 overexpression-mediated phenotypes of CRC cell proliferation and migration in vitro. (A, B) Cell proliferation was assessed by CCK-8 assay and flow cytometry cell-cycle analysis in CASC11 overexpression RKO and SW620 cells. Error bars represent the means ± SD of 3 independent experiments. (C, D) Cell migration was analyzed by scratch wound assay and Transwell migration assay in CASC11 overexpression RKO and SW620 cells. Error bars represent the means ± SD of 3 independent experiments or 5 different fields, respectively. *p < 0.05; **p < 0.01; ***p < 0.001. [file Image_2.tif]

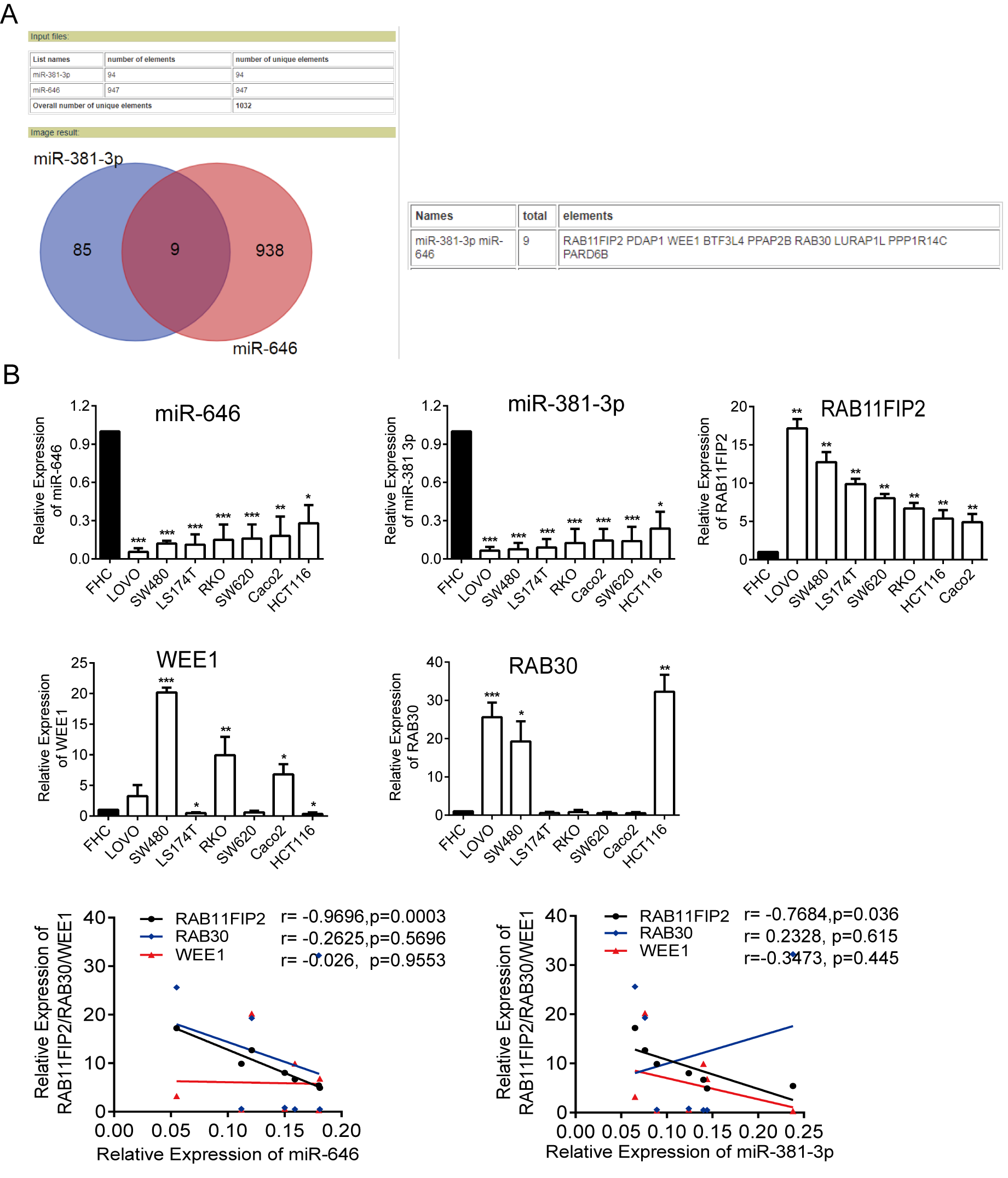

Supplement: Supplementary Figure 3 — Selecting RAB11FIP2 as a mutual target gene of miR-646 and miR-381-3p. (A) Venn diagrams showing the intersection set of mutual target genes of miR-646 and miR-381-3p. (B) qRT-PCR assays showed expressions of RAB11FIP2, WEE1, RAB30, miR-646, and miR-381-3p in 8 cell lines. Spearman correlation analysis showed a negative relationship between RAB11FIP2 mRNA and the above two miRNAs. Error bars indicate the means ± SD of 3 independent experiments. *p < 0.05; **p < 0.01; ***p < 0.001. [file Image_3.tif]

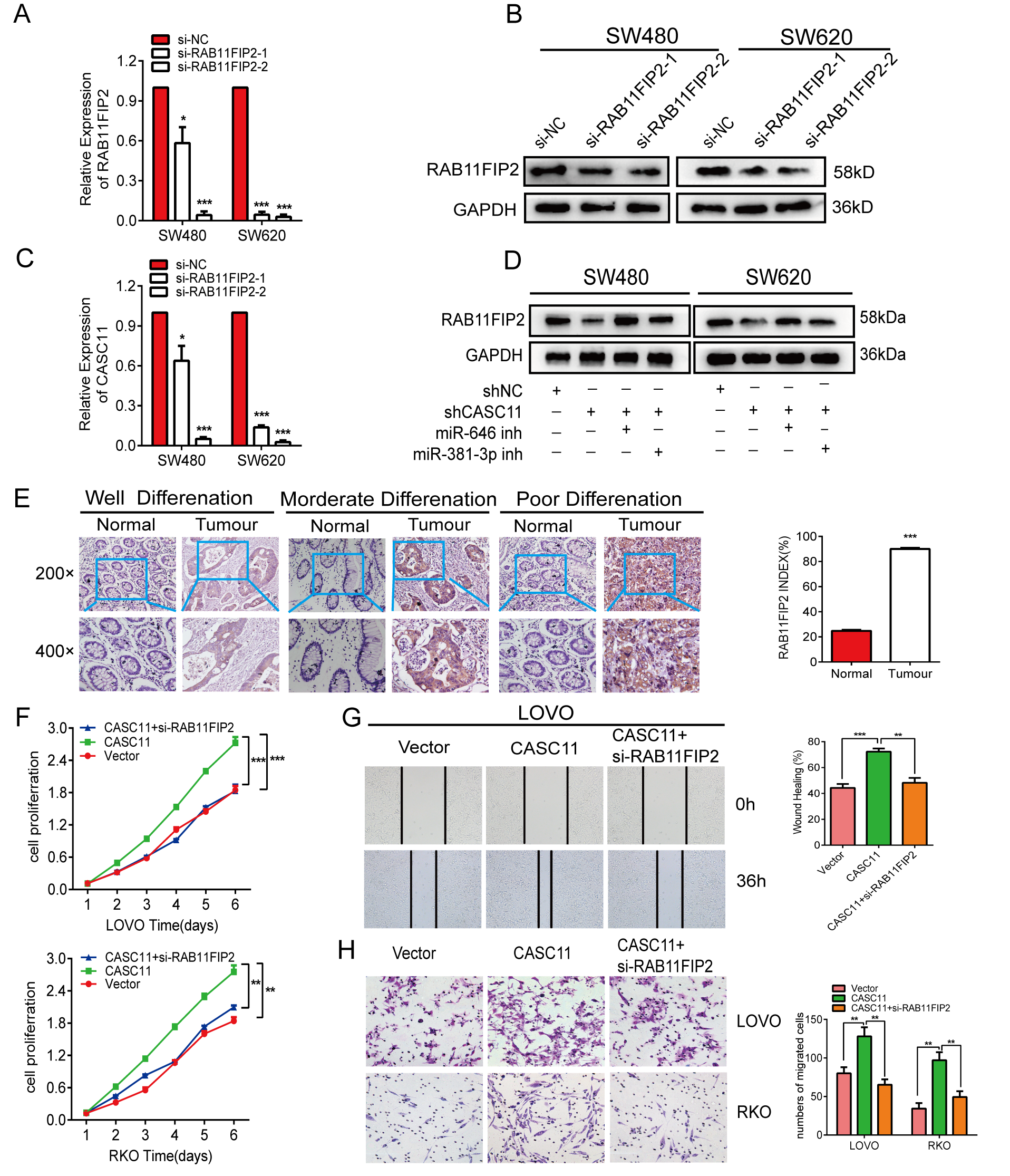

Supplement: Supplementary Figure 4 — RAB11FIP2 positively regulated CASC11 expression in CRC cells, and RAB11FIP2 knockdown can prohibit CRC cell proliferation and migration mediated by CASC11 overexpression. (A, B) The successful construction of siRAB11FIP2 cells was confirmed by qRT-PCR and WB. (C) The relative expressions of CASC11 in CRC cells transfected with siRAB11FIP2 by qRT-PCR. (D) miR-646 and miR-381-3p inhibitors rescued a decrease in RAB11FIP2 protein levels caused by CASC11 knockdown.(E) Immunohistochemical staining (IHC) evaluation of the expression of RAB11FIP2 in paraffin-embedded human CRC tissues and adjacent normal tissues (n=67). The error bars in all graphs represent the means ± SD of 3 different fields. (F–H) siRAB11FIP2 reverses CASC11 overexpression-mediated phenotypes of CRC cell proliferation and migration in vitro. Cell proliferation was assessed by CCK-8 assay, and cell migration was analyzed by scratch wound assay and Transwell migration assay. *p < 0.05; **p < 0.01; ***p < 0.001. [file Image_4.tif]

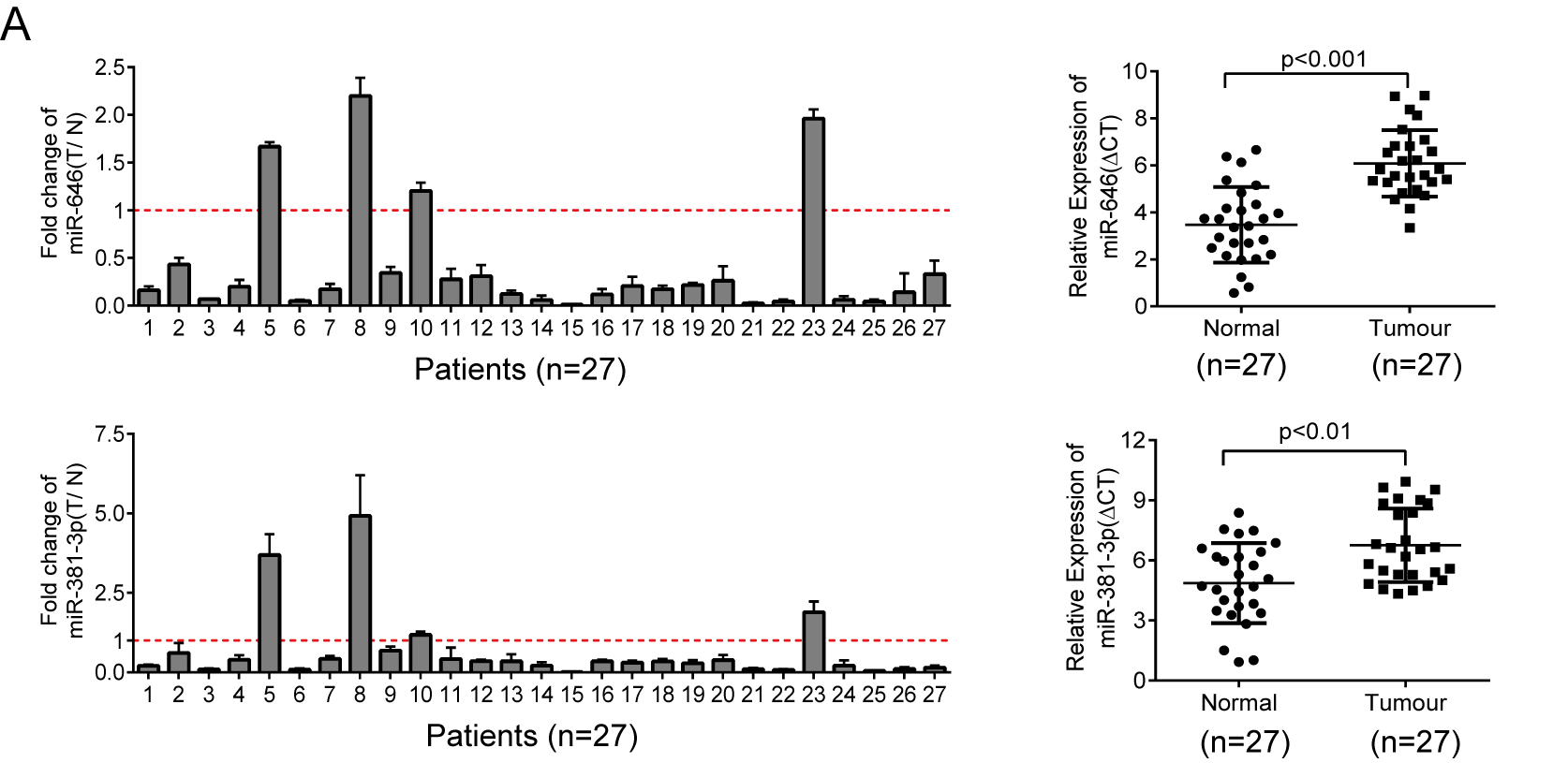

Supplement: Supplementary Figure 5 — (A) qRT-PCR analysis of miR-646 and miR-381-3p expression in 27 paired CRC tissues. Left panel, the results were presented as the fold change in tumour tissues relative to the matched adjacent normal tissues. Right panel, comparison of miR-646 and miR-381-3p expression in 27 paired CRC tissues (T) and matched normal tissues (N). Error bars indicate the means ± SD of 3 independent experiments. [file Image_5.tif]
