# Supplementary material for: The LncRNA CASC11 Promotes Colorectal Cancer Cell Proliferation and Migration by Adsorbing miR-646 and miR-381-3p to Upregulate Their Target RAB11FIP2
Source: Front Oncol. 2021 Apr 15;11:657650. doi: 10.3389/fonc.2021.657650 (PMC8084185; doi:10.3389/fonc.2021.657650)
Supplement: Supplementary file 6 [file DataSheet_1.pdf]

Table S1 Clinicopathologic characteristics of CASC11 and RAB11FIP2 expression in CRC patients.

Clinicopathological Variables	n	CASC11		X^2	P	RAB11FIP2		X^2	P
		High expression	Low expression			High expression	Low expression		
Age(years) [a]									
≤50	17	8	9	0.422	0.695	8	9	0.022	1.000
>50	10	6	4			5	5		
Gender									
Male	14	7	7	0.040	1.000	7	7	0.040	1.000
Female	13	7	6			6	7		
Tumour Size (cm) [b]									
≤3.25	20	7	13	8.775	0.006	7	13	5.342	0.033
>3.25	7	7	0			6	1		
Differentiation									
Well/Moderate	20	9	11	1.451	0.385	9	11	0.306	0.678
Poor	7	5	2			4	3		
Lymph-Vascular Invasion									
Yes	8	7	1	5.787	0.033	6	2	3.283	0.103
No	19	7	12			7	12		
Lymph Metastasis									
Yes	11	9	2	6.677	0.018	8	3	4.492	0.054
No	16	5	11			5	11		
T-Stage									
T1-2	9	1	8	8.975	0.004	1	8	7.418	0.013
T3-4	18	13	5			12	6		

[a] Group of age was performed according to median.

[b] Tumour size was grouped according to median.
